# LimeMap: a comprehensive map of lipid mediator metabolic pathways

**DOI:** 10.1038/s41540-020-00163-5

**Published:** 2021-01-27

**Authors:** Akinori Nishi, Katsuya Ohbuchi, Noriko Kaifuchi, Chika Shimobori, Hirotaka Kushida, Masahiro Yamamoto, Yoshihiro Kita, Suzumi M. Tokuoka, Ayako Yachie, Yukiko Matsuoka, Hiroaki Kitano

**Affiliations:** 1 Tsumura Kampo Research Laboratories, Tsumura & Co., Ibaraki, Japan; 2 Life Sciences Core Facility, Graduate School of Medicine, The University of Tokyo, Tokyo, Japan; 3 Department of Lipidomics, Graduate School of Medicine, The University of Tokyo, Tokyo, Japan; 4 The Systems Biology Institute, Shinagawa, Tokyo Japan

**Keywords:** Immunology, Biochemical networks, Software

## Abstract

Lipid mediators are major factors in multiple biological functions and are strongly associated with disease. Recent lipidomics approaches have made it possible to analyze multiple metabolites and the associations of individual lipid mediators. Such systematic approaches have enabled us to identify key changes of biological relevance. Against this background, a knowledge-based pathway map of lipid mediators would be useful to visualize and understand the overall interactions of these factors. Here, we have built a precise map of lipid mediator metabolic pathways (LimeMap) to visualize the comprehensive profiles of lipid mediators that change dynamically in various disorders. We constructed the map by focusing on ω-3 and ω-6 fatty acid metabolites and their respective metabolic pathways, with manual curation of referenced information from public databases and relevant studies. Ultimately, LimeMap comprises 282 factors (222 mediators, and 60 enzymes, receptors, and ion channels) and 279 reactions derived from 102 related studies. Users will be able to modify the map and visualize measured data specific to their purposes using CellDesigner and VANTED software. We expect that LimeMap will contribute to elucidating the comprehensive functional relationships and pathways of lipid mediators.

## Introduction

Lipid mediators derived from fatty acids (FAs) comprise a major group of bioactive metabolites. They are produced by lipid-metabolizing enzymes in vivo and function through specific G protein-coupled receptors (GPCRs). Prostaglandins (PGs), leukotrienes (LTs), and hydroxyeicosatetraenoic acids (HETEs), which are metabolites of arachidonic acid (AA) and ω-6 polyunsaturated FA (PUFA), are classic lipid mediators and form a family of bioactive lipids called “eicosanoids”. Enzymes involved in eicosanoid metabolism have been extensively studied, and the metabolic pathway is referred to as the AA cascade. In addition, GPCRs for eicosanoid lipid mediators have been mostly identified and characterized^1^.

Accumulating evidence has revealed the roles of eicosanoids in various pathological and physiological responses, including inflammatory response, vasodilation, platelet aggregation, asthma, fever, pain, and cancer^1–8^. As a result, the AA cascade is recognized as an effective drug target. For example, nonsteroidal anti-inflammatory drugs including aspirin that targets cyclooxygenases (COXs), key enzymes of PG biosynthesis, are some of the most widely used drugs; drugs that target LT receptors (e.g. Pranlukast) are used as antiallergy therapies; and PGE1-derivatives and PGF_2α_ are used as anti-platelet/vasodilators and labor-inducing drugs, respectively^9–12^.

In addition to the AA cascade, recent studies have extended the repertoire of lipid mediators to ω-3 PUFA, for example, eicosapentaenoic acid (EPA) and docosahexaenoic acid (DHA) are oxidized in a similar manner to AA, producing various anti-inflammatory and pro-resolving mediators such as resolvins and protectins^13–17^.

Research on the biological roles of lipid mediators has advanced through the identification and functional analysis of protein factors, such as lipid mediator-producing enzymes and lipid mediator receptors. Studies using genetically modified animals have revealed various pathophysiological roles of individual lipid mediators. However, because lipid mediators share precursors and biosynthetic pathways and often have functional interactions, it is clear that a comprehensive analysis covering a large number of lipid mediators is necessary to uncover their diverse roles in vivo.

Recent improvements to mass spectrometers in terms of speed, sensitivity, and selectivity have accelerated the development of strategies for the comprehensive analysis of lipid mediators, which were previously assessed via single-target analyses such as an enzyme-linked immunosorbent assay^18–20^. Currently, more than a hundred lipid mediator-related metabolites can be simultaneously quantified in a single sample, and the resulting data can be assessed by multivariate analyses such as principal component analysis (PCA), partial least squares regression (PLS) or PLS-discriminant analysis (PLS-DA), and clustering analysis^21,22^. Analyses based on the quantitative profiles of lipid mediators can identify those that are significant for specific biological functions, and can also reveal functional similarities or reciprocity among different lipid mediators. Without a pathway-based interpretation, however, the analysis sometimes fails to demonstrate biological meaning. Integrating pathway information with metabolite quantification data is an effective metabolomics strategy: as lipid mediators and enzymes (i.e., regulatory factors) are linked on the metabolic pathway map, so a comprehensive overview of the biological processes becomes apparent. To this end, there are growing demands for resources and tools that can disentangle a complex omics dataset as well as visualize it. For general metabolomics and lipidomics purposes, public databases such as the Kyoto Encyclopedia of Genes and Genomes (KEGG) (http://www.kegg.jp)^23^, PANTHER (http://www.pantherdb.org)^24^, LIPID MAPS (http://www.lipidmaps.org)^25^, Human Metabolome Database (HMDB) (http://www.hmdb.ca)^26^, and PubChem (https://pubchem.ncbi.nlm.nih.gov) have become essential research resources. There also are tools that are more domain- or interest-specific, aimed for the visualization of specific types of omics data on biological pathway networks^27–35^. At present, however, there is no pathway map with sufficient accuracy and coverage for visualizing lipid mediator datasets. The aim of the present study was, therefore, to fill this gap. Lipid Mediator Metabolic Pathway Map (LimeMap), which maps lipid mediators and related metabolites of ω3-FA and ω6-FA origin onto the metabolic pathway with relevant enzymes and receptors, has been built to simultaneously visualize and annotate experimental data obtained from widely-targeted mediator lipidomics analysis. It integrates information from manually curated literature, public knowledge bases, and databases. To demonstrate its application, we measured lipid mediators in a mouse model of acute inflammation and mapped them onto LimeMap, implemented in CellDesigner- and VANTED-compatible formats. We demonstrate that LimeMap can be used to effectively visualize datasets derived from comprehensive lipid mediator analysis.

## Results and discussion

### Development and characteristics of LimeMap

**Fig. 1:**
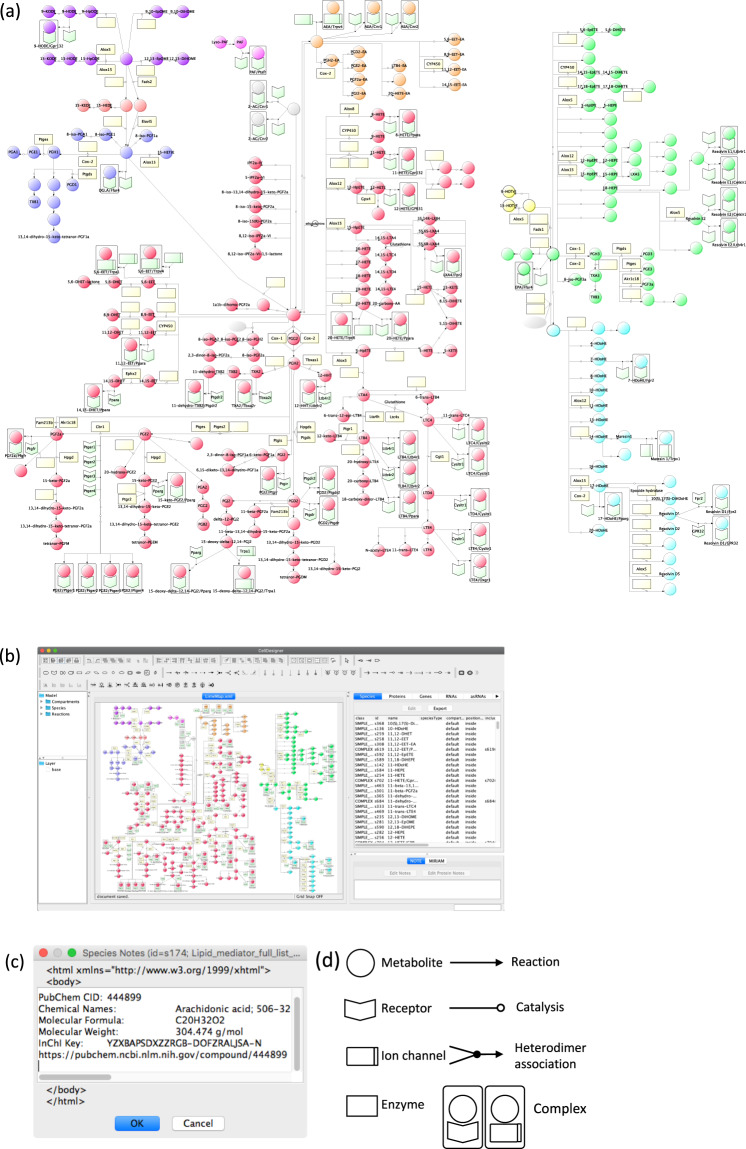
Lipid mediator metabolic pathway map (LimeMap). **a** LimeMap includes a total of 282 factors (222 lipid mediators and metabolites, 60 enzymes and receptors) and 279 reactions. The LimeMap file, SBML compliant format created with CellDesigner version 4.4, is available as Additional Data (LimeMap.xml). The list of lipid metabolite abbreviations is provided in Supplementary Table 1. The magnified pathway maps are shown in Supplementary Fig. 1. **b** The SBML file is opened in CellDesigner. **c** Information on each factor is annotated in the notes section. **d** Definition of the symbols used to build the map. Circle symbols show metabolites, square symbols in light yellow show enzymes, arrowhead symbols show receptors, and separated square symbols show ion channels. Complexes of metabolite and receptor or ion channel are indicated with a grey-colored square. Lines with an arrow show metabolic reactions, lines with an open circle connected to a metabolic reaction show enzymatic reactions, and arrowhead symbols with two connected lines show heterodimer association. Color coding indicates the grouping of metabolites; Red for AA derived lipid mediators, purple for LA derived lipid mediators, salmon pink for GLA and EDA derived lipid mediators, blue for DGLA derived lipid mediators, pink for Lyso-PAF and PAF, orange for AEA derived lipid mediators, yellow for ALA derived lipid mediator, light green for EPA derived lipid mediator, and light blue for DHA derived lipid mediators.

We built LimeMap by integrating current knowledge of the metabolic pathways of lipid mediators via the manual curation of related studies and databases, such as the Alliance of Genome Resources, HMDB, KEGG, LIPID MAPS, and PubChem. The relationships among metabolic processes were constructed by using CellDesigner software in a standard Systems Biology Markup Language (SBML) format, and visualized as a Systems Biology Graphical Notation (SBGN) diagram^33^. The files generated by the software in XML format (LimeMap.xml)^33^ can be flexibly edited and expanded with user information and data. By integration of current knowledge, LimeMap can support researchers in a comprehensive analysis of the interactions of lipid mediators (Fig. 1)^33^. Ultimately, LimeMap.xml comprises 282 factors (222 lipid mediators and metabolites in addition to 60 enzymes and receptors) and 279 reactions derived from 102 related studies, each of which is annotated in the “notes” section for each node, link, enzyme and receptor. The map has also been ported to VANTED software in Geography Markup Language (GML) format (LimeMap.gml)^34^. LimeMap in both files are available as LimeMap.xml/ LimeMap.gml. The initial version of LimeMap reported in this study mainly focuses on ω6- and ω3- FA metabolites even though there are several other classes of bioactive lipids, such as lysophospholipids which also have important roles. Both of the ω6- and ω3 lipid mediator pathways are critically important and interrelate to control biological functions such as initiation and resolution of the inflammatory response^16^. The metabolites derived from AA: PGs, LTs, HETEs, and EETs, which form a family of bioactive lipids called eicosanoids, are included in the map. The map also includes ω3-FA metabolites, resolvins and maresins, which are essential in anti-inflammatory processes and resolving inflammation. Furthermore, we used mouse gene symbols to describe enzymes and receptors which are related to lipid mediators in the map. Although human and mouse lipid mediator pathways are thought to be generally conserved, they are known to differ for some factors. For instance, part of the role of lipoxygenases differs between human and mouse^36^: expression of human ALOX15 mainly produces a 15-lipoxygenating activity while mouse Alox15 expression mainly produces 12-HpETE. Human ALOX15B produces 15-HpETE from AA and, in contrast, mouse Alox15b has arachidonic acid 8-lipoxygenating activity. Therefore, species differences should be noted.

LimeMap can be used with CellDesigner software and VANTED software on the GARUDA platform (http://www.garuda-alliance.org/).

### Analysis of the lipid mediator profile in a mouse model of acute inflammation

**Fig. 2:**
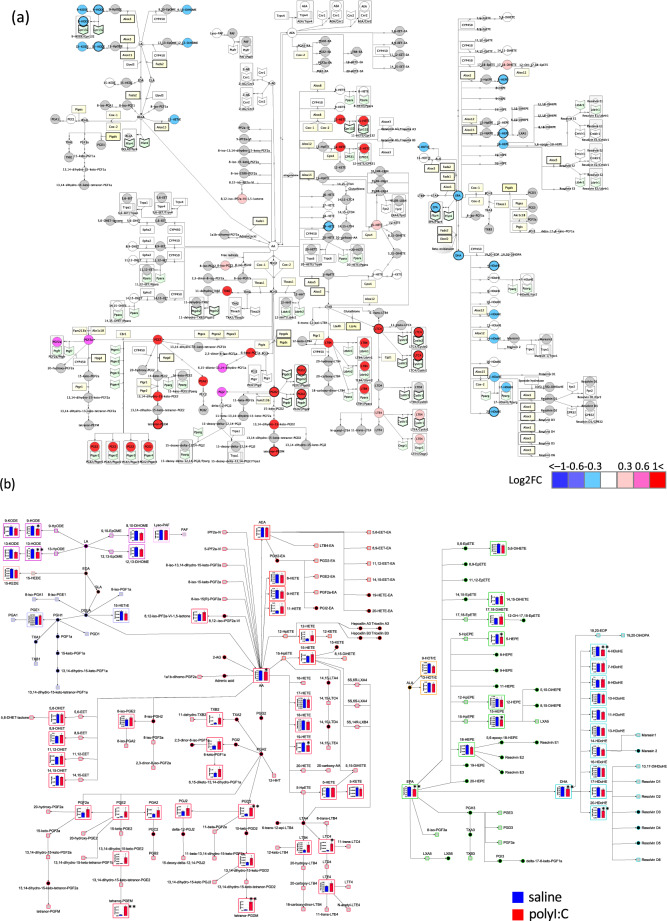
Pathway map of measured changes in lipid mediators between control and polyI:C treatment: SBML format via CellDesigner; GML format via VANTED: effect of polyI:C treatment on lipid mediators in plasma. **a** Fold changes in lipid mediators induced by polyI:C versus control treatment, mapped in SBML format using CellDesigner. Metabolites not included and not detected are shown as small grey circles and larger grey circles, respectively. Metabolites showing a log2 fold change (polyI:C/saline) > 0.3 or < -0.3 (*P* < 0.05) are drawn with a thick outline; related enzymes/receptors are highlighted. Lines with an arrow show a metabolic reaction; lines with an open circle, connected to a metabolic reaction, show an enzymatic reaction. The receptor, ion channel, and enzymes associated with the pathway of changed lipid mediator profile is indicated by light green and light yellow symbols, respectively. The receptor, ion channel, and enzyme associated with the pathway of significantly changed lipid mediator between polyI:C and saline are drawn with a thick outline. **b** Mean (± s.e.m) amounts of lipid mediator measured in plasma after saline (blue column) and polyI:C (red column) treatment were mapped in GML format using VANTED. Metabolites not included and not detected in the analysis are shown as small black circles and grey squares, respectively. Lines with arrows show a metabolic reaction. Colors indicate the metabolite grouping for lipid derived mediators: red; AA, purple; LA, salmon pink; GLA and EDA, blue; DGLA, pink; Lyso-PAF and PAF, orange; AEA, yellow; ALA, light green; EPA, and light blue; DHA. **P < 0.01, *P < 0.05.

To demonstrate the application of LimeMap, we carried out a comparative analysis of lipid mediator profiles in normal mice versus a model of acute inflammation induced by a synthetic double-stranded RNA, polyinosinic-polycytidylic acid (polyI:C)^37–39^. Mice were intraperitoneally injected with polyI:C or vehicle, and after 2 h, plasma was collected and analyzed for lipid mediator profiles using liquid chromatography tandem mass spectrometry (LC-MS/MS)^19^. The profile data will provide important information on the inflammatory responses of lipid mediators; however, it is important to consider the sampling conditions, such as anesthesia, when comparing the profile data between different experiments. We measured a total of 158 metabolites, (Supplementary Table 2), and 66 of these were detected in plasma. The signal intensities of the metabolites (normalized to an internal standard) are summarized in Supplementary Table 3. Of the 158 metabolites, all except oleoylethanolamine, azelaoyl-PAF, PGE1-EA, PGK2, and 7,17-hydroDPA were mapped in LimeMap. To elucidate the metabolic pathways affected by polyI:C treatment, we visualized the fold changes of lipid mediators on LimeMap using CellDesigner software (Fig. 2a). We also mapped the quantities of lipid mediators on LimeMap using VANTED software (Fig. 2b). The maps in Fig. 2 successfully illustrate the effects of polyI:C treatment on lipid mediator metabolic pathways in plasma. The data from the plasma samples projected on LimeMap clearly revealed an overall increase in AA-derived metabolites which is in contrast to the decreases in LA-derived, EPA-derived, and DHA-derived metabolites (Fig. 2a, b). Again, the map visualization enabled us to identify active pathways: PGD2 and its metabolites, as well as PGE2 and its metabolites, were clearly increased by polyI:C treatment (Fig. 2a). It should be noted that not all changes were statistically significant (Supplementary Table 3); nevertheless, the pathway-based evaluation provides more comprehensive and intuitive information required to understand the biological function and biomarkers associated with inflammation related diseases, such as infectious diseases, when compared with a metabolite-wise comparison^40–42^.

In this manner, LimeMap is useful as a means for comprehensive comparison of lipid mediator profiles.

In summary, we have developed LimeMap as a tool to facilitate comprehensive analysis of lipid mediator data. Users can modify the map and visualize measured data for their purposes via CellDesigner and VANTED software. At present, coverage of the map is limited to what we curated and what we knew. A continuous effort to curate and extend the pathways based on feedback, correction and the most recent data from the research community will be necessary to develop the map and keep it updated, and to further enhance its usability.

As a future project for using LimeMap, kinetic laws regarding the metabolic reactions can be assigned to the map in SBML format; in addition, we are planning to make a simulation model. Lastly, we are planning to accumulate lipid mediator profiles in specific tissues and under various physiological conditions such as aging, and are trying to reveal the functional networks of lipid mediators using the LimeMap pathways^43,44^.

## Methods

### Construction of LimeMap

The metabolites in LimeMap mainly include those described by Yamada et al,^19^ who developed a method for the simultaneous analysis of major lipid mediators. In addition, to describe the metabolic pathways, we manually curated information on lipid mediators, their metabolic interactions, and factors related to metabolism and function such as enzymes and receptors/ion channels, as described in both public databases [Alliance of Genome Resources (http://www.alliancegenome.org), Human Metabolome Database HMDB (http://www.hmdb.ca)^26^, KEGG^23^, LIPID MAPS, and PubChem] and published manuscripts, as noted in the “notes” section of the map. The basic LimeMap was built mainly through the use of CellDesigner version 4.4 software. The map conforms to SBML, a data exchange format based on XML, and is represented in the graphical notation of CellDesigner, which adheres to the standards of SBGN. The map content, factors, and reactions were produced using an SBGN process description language, which facilitates the visualization of state transitions.

For map construction, each lipid mediator was described as a simple molecule and constituted the edge of the pathway. Information, such as molecular formula, molecular weight, chemical name, and InChI Key, was mainly collected from the PubChem database and included in the notes section for each lipid mediator. The molecules were connected in a stepwise manner in accordance with the metabolic process. Metabolic enzymes, described by their mouse gene symbol, were connected to the nodes as catalytic factors. Furthermore, receptors associated with specific lipid mediators were placed in the map as related lipid mediators. Both enzymes and receptors were described mainly by their mouse gene symbol, referenced from the Alliance of Genome Resources (http://www.alliancegenome.org), while the gene name, synonyms, and human and rat orthologous genes were included the notes section. To enable visualization of the measured value by several graphical methods, we also built a map in GML format using VANTED software based on the XML-formatted LimeMap.

### Analysis of the lipid mediator profile in a mouse model of acute inflammation

#### Animals

Male C57BL/6 J mice were purchased from Charles River Laboratories International, Inc. (Kanagawa, Japan) and used for lipid profiling at 8 weeks of age after habituation. Mice were housed individually in a cage with paper chips, and permitted free access to food and water. The rearing conditions were room temperature 23 °C, relative humidity 60%, and 12 h light-dark cycle (7:00-19:00). Mice were maintained and used for the experiments in accordance with the Guidelines for the Care and Use of Laboratory Animals of Tsumura & Co. Before the experiment, the average body weight was matched between groups and used. All experimental procedures were carried out upon approval from the Laboratory Animal Committee of Tsumura & Co., and performed in accordance with guidelines for the conduct of animal experiments in ministry of health, labour and welfare, Japan.

#### Administration of polyI:C

Six milligrams of polyI:C supplied by InvivoGene [PolyI:C(HMW), San Diego, CA, USA] was dissolved in 10 ml of saline (Otsuka Pharmaceutical Co, Tokyo, Japan) by heating the mixture for 10 min at 70 °C. The solution was cooled for 1 h at room temperature to ensure proper annealing before administration to mice by intraperitoneal injection at a dose of 6 mg/kg. polyI:C was administered to nine mice, and saline was administered to ten mice as the control group. We did not consider the blinding. Distilled water was given to all mice at the same time as the injection.

#### Blood sampling

Two hours after treatment with polyI:C, mice were anesthetized with isoflurane (AbbVie Inc., North Chicago, IL, USA), and blood was collected from the inferior vena cava with EDTA.2 K (Wako Pure Chemical Industries, Osaka, Japan). Blood samples were centrifuged at 4 °C to prepare plasma samples, which were stored at −80 °C until use.

#### Analysis of lipid mediators by LC-MS/MS

Lipid mediators were measured in mouse plasma sampled 2 h after treatment with polyI:C. To extract low molecular weight metabolites for LC/MS analysis, 1 ml of methanol containing a mixture of internal standards (0.5 ng/μl each of tetranor-PGEM-d6, TXB2-d4, PGE2-d4, PGD2-d4, LTC4-d5, LTB4-d4, 5-HETE-d8 and 15-HETE-d8; 0.25 ng/μl of oleoylethanolamide-d4; and 10 ng/μl of AA-d8; all from Cayman Chemical, Ann Arbor, MI, USA) was mixed with 100 μl of plasma sample for 5 min at room temperature and then centrifuged at 15,000 *g* for 3 min. The supernatant was diluted with 4 ml of 0.1% formic acid in water and gently mixed. The mixture was loaded onto a preconditioned solid-phase extraction cartridge (STRATA-X, 10 mg/1 ml, Phenomenex, Torrance, CA, USA), which was washed with 1 ml of 0.1% formic acid, followed by 1 ml of 15% ethanol. The lipids were eluted with 250 μl of 0.1% formic acid in methanol; the eluent was then reduced by vacuum evaporator and reconstituted in 20 μl of methanol. Five microliters of sample were used for LC/MS analysis. The LC/MS system consisted of two LC-30AD pumps, a SIL-30AC auto-sampler, a CTO-20A column oven, a CBM-20A system controller, and a triple-quadrupole mass spectrometer LCMS-8050 (Shimadzu). A reversed-phase column (Kinetex C8, 2.1 × 150 mm, 2.6 μm, Phenomenex) was used for chromatographic separation. Chromatogram acquisition, detection of mass spectral peaks, and waveform processing were performed using LCMS solution software and the LC-MS/MS Method Package for Lipid Mediators version 2 (Shimadzu), which contains method files specifying the analytical conditions, and data analysis parameters for 158 lipid mediators derived from AA, EPA or DHA, among others. The peak area of each quantified ion was calculated and normalized to those of the internal standards. For normalization, the intensity of detected peaks was divided by the peak intensity of the standard in the same category. Processing of metabolomics data was performed with Excel software (Microsoft Corporation, Redmond, WA, USA). Missing values in the raw data were replaced by half of the minimum positive value, and these data were used for subsequent statistical analysis.

#### Statistical analysis

Statistical analysis was conducted using Excel software. Statistical significance was tested by two-sided Welch’s *t*-test, and differences with *P* < 0.05 were considered significant.

#### Data visualization in LimeMap

The measured data were visualized in LimeMap using CellDesigner and VANTED software on the GARUDA platform (http://www.garuda-alliance.org)^45^.

## Supplementary information

Supplementary information

Supplementary Data

Supplementary Data

## Data Availability

Metabolomics data measured by LC-MS/MS are shown in Supplementary Tables 3. Relevant experimental data are available from the authors.

## Code availability

The LimeMap.xml and LimeMap.gml files are attached.
