# Publisher Correction: LimeMap: a comprehensive map of lipid mediator metabolic pathways

**DOI:** 10.1038/s41540-021-00174-w

**Published:** 2021-03-08

**Authors:** Akinori Nishi, Katsuya Ohbuchi, Noriko Kaifuchi, Chika Shimobori, Hirotaka Kushida, Masahiro Yamamoto, Yoshihiro Kita, Suzumi M. Tokuoka, Ayako Yachie, Yukiko Matsuoka, Hiroaki Kitano

**Affiliations:** 1 Tsumura Kampo Research Laboratories, Tsumura & Co., Ibaraki, Japan; 2 Life Sciences Core Facility, Graduate School of Medicine, The University of Tokyo, Tokyo, Japan; 3 Department of Lipidomics, Graduate School of Medicine, The University of Tokyo, Tokyo, Japan; 4 The Systems Biology Institute, Shinagawa, Tokyo, Japan

**Keywords:** Immunology, Biochemical networks, Software

Correction to: *npj Systems Biology and Applications* 10.1038/s41540-020-00163-5.

The original version of the published Article contained an incorrect version of the Supplementary Information. This has been corrected in the HTML version of the Article.

