# Supplementary material for: LimeMap: a comprehensive map of lipid mediator metabolic pathways
Source: NPJ Syst Biol Appl. 2021 Jan 27;7:6. doi: 10.1038/s41540-020-00163-5 (PMC7840682; doi:10.1038/s41540-020-00163-5)
Supplement: Supplementary file 1 — Supplementary information [file 41540_2020_163_MOESM1_ESM.pdf]

LimeMap: a comprehensive map of lipid mediator metabolic pathways

Akinori Nishi[1], Katsuya Ohbuchi[1], Noriko Kaifuchi[1], Chika Shimobori[1], Hirotaka Kushida[1], Masahiro Yamamoto[1], Yoshihiro Kita[2,3], Suzumi M. Tokuoka[3], Ayako Yachie[4], Yukiko Matsuoka[4], Hiroaki Kitano[4]

1; Tsumura Kampo Research Laboratories, Tsumura & Co., Ibaraki, Japan.

2; Life Sciences Core Facility, Graduate School of Medicine, The University of Tokyo, Tokyo, Japan.

3; Department of Lipidomics, Graduate School of Medicine, The University of Tokyo, Tokyo, Japan.

4; The Systems Biology Institute, Shinagawa, Tokyo, Japan.

Supplementary Figure 1

Lipid mediator metabolic pathway map (LimeMap)

a) LA, EDA, DGLA and AEA derived lipid mediators, b) AA derived lipid mediators, and c) EPA and DHA derived lipid mediators

Supplementary Table 1

List of abbreviations of the lipid metabolites shown in Fig. 1a

Supplementary Table 2

List of measured metabolites

Of the 158 metabolites, 153 were included in the map. Oleoylethanolamide (OEA), azelaoyl-platelet activating factor (azelaoyl-PAF), PGE1-EA, PGK2, and 7,17-hydrozy-docosapentaenoic acid (7,17-hydrozy-DPA) were not included in the map.

Supplementary Table 3

Metabolites detected in plasma

Supplementary Data

LimeMap.xml

The files generated by CellDesigner software in XML format

LimeMap.gml

The files generated by VANTED software in GML format

Supplementary Figure 1

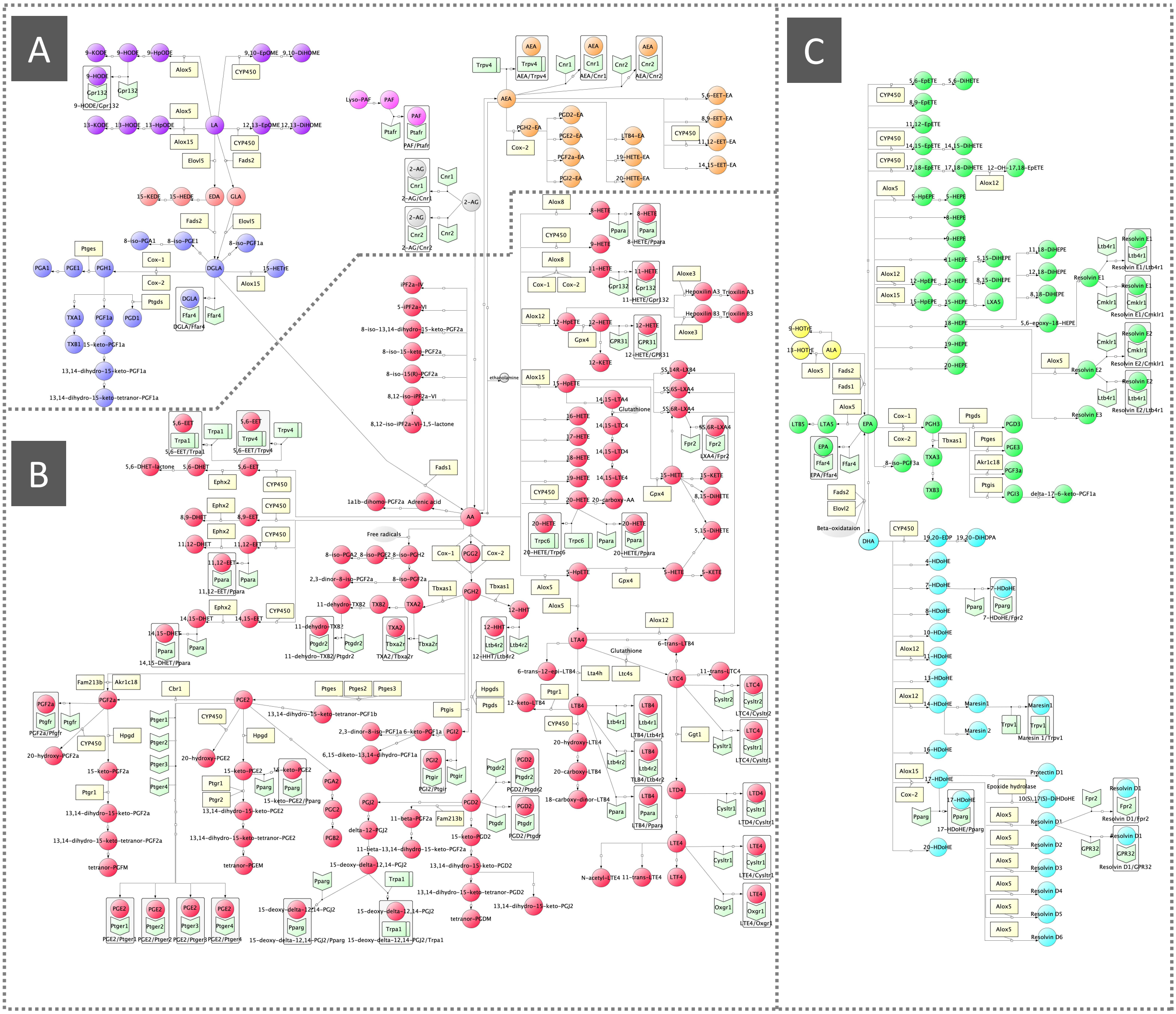

Supplementary Figure 1

A

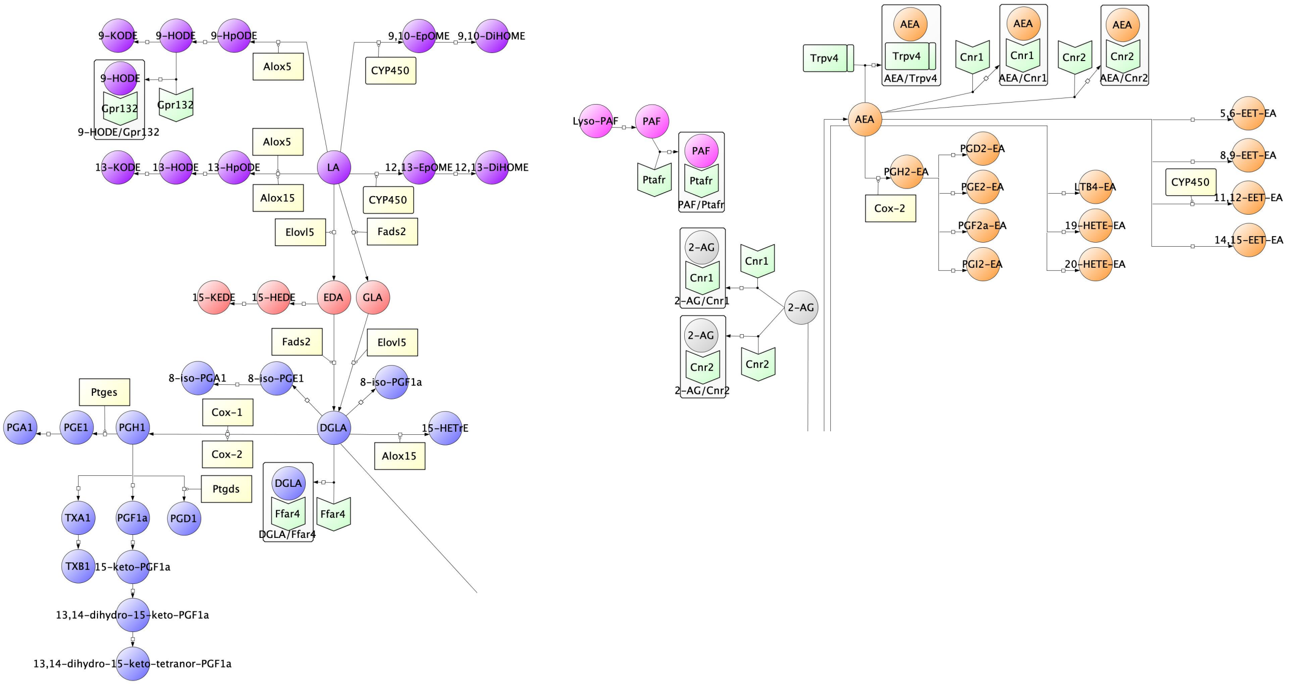

Supplementary Figure 1

B

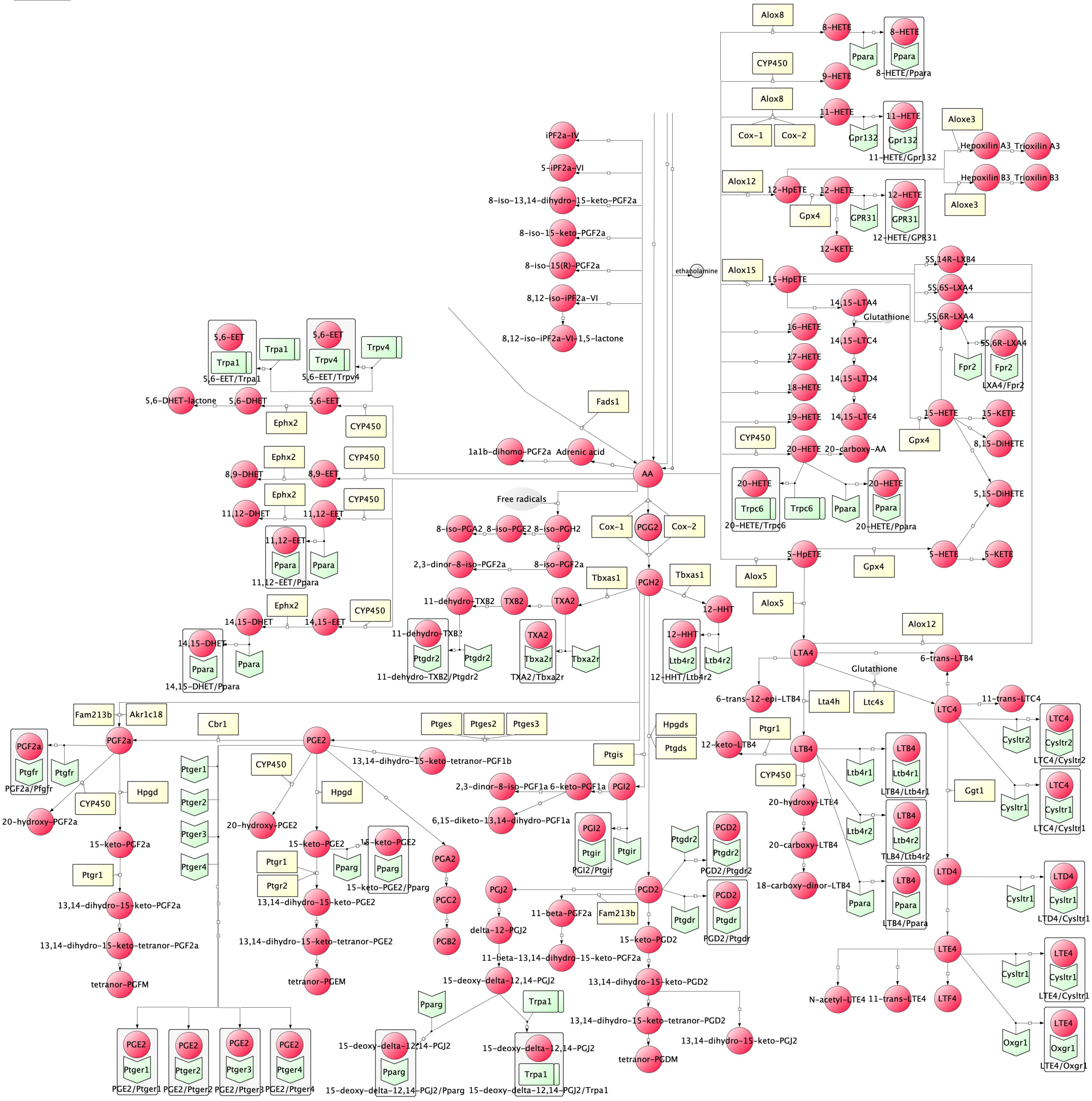

Supplementary Figure 1

C

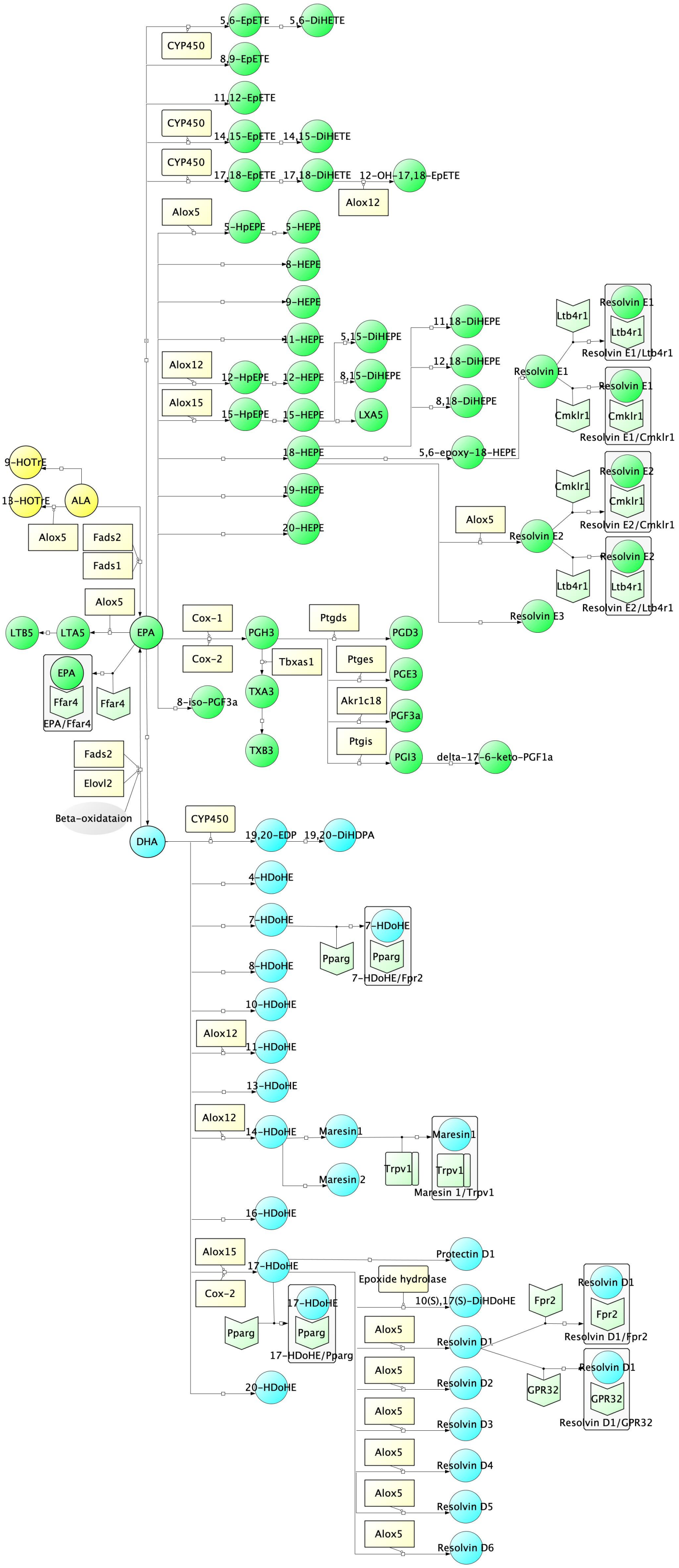

Supplementary Table 1

Abbreviation	Name
AA	arachidonic acid
Adrenic acid	adrenic acid
AEA	anandamide
ALA	alpha linolenic acid
delta-12-PGJ2	delta-12-prostaglandin J2
delta-17-6-keto-PGF1a	delta-17-6-keto-prostaglandin F1alpha
DGLA	dihomo gamma linolenic acid
DHA	docosahexaenoic acid
EDA	eicosadienoic acid
EPA	eicosapentaenoic acid
GLA	gamma linolenic acid
Hepoxilin A3	hepoxilin A3
Hepoxilin B3	hepoxilin B3
iPF2a-IV	isoprostane F2 alpha-IV
LA	linoleic acid
LTA4	leukotriene A4
LTA5	leukotriene A5
LTB4	leukotriene B4
LTB4-EA	leukotriene B4 ethanolamide
LTB5	leukotriene B5
LTC4	leukotriene C4
LTD4	leukotriene D4
LTE4	leukotriene E4
LTF4	leukotriene F4
LXA5	lipoxin A5
Lyso-PAF	lyso-platelet-activating factor
Maresin 2	maresin 2
Maresin1	maresin 1
N-acetyl-LTE4	N-acetyl leukotriene E4
PAF	platelet-activating factor
PGA1	prostaglandin A1
PGA2	prostaglandin A2
PGB2	prostaglandin B2
PGC2	prostaglandin C2
PGD1	prostaglandin D1
PGD2	prostaglandin D2
PGD2-EA	prostaglandin D2 ethanolamide
PGD3	prostaglandin D3
PGE1	prostaglandin E1
PGE2	prostaglandin E2
PGE2-EA	prostaglandin E2 ethanolamide
PGE3	prostaglandin E3
PGF1a	prostaglandin F1alpha
PGF2a	prostaglandin F2alpha
PGF2a-EA	prostaglandin F2alpha
PGF3a	prostaglandin F3alpha
PGG2	prostaglandin G2
PGH1	prostaglandin H1
PGH2	prostaglandin H2
PGH2-EA	prostaglandin H2 ethanolamide
PGH3	prostaglandin H3
PGI2	prostaglandin I2
PGI2-EA	prostaglandin I2 ethanolamide
PGI3	prostaglandin I3
PGJ2	prostaglandin J2
Protectin D1	10R,17S-dihydroxy-docosahexaenoic acid
Resolvin D1	resolvin D1
Resolvin D2	resolvin D2
Resolvin D3	resolvin D3
Resolvin D4	resolvin D4
Resolvin D5	resolvin D5
Resolvin D6	resolvin D6
Resolvin E1	resolvin E1
Resolvin E2	resolvin E2
Resolvin E3	resolvin E3
tetranor-PGDM	tetranor-prostaglandin D metabolite
tetranor-PGEM	tetranor-prostaglandin E metabolite
tetranor-PGFM	tetranor-prostaglandin F metabolite
Trioxilin A3	Trioxilin A3
Trioxilin B3	Troxilin B3
TXA1	thromboxane A1
TXA2	thromboxane A2
TXA3	thromboxane A3
TXB1	thromboxane B1
TXB2	thromboxane B2
TXB3	thromboxane B3
1a1b-dihomo-PGF2a	1a,1b-dihomo-prostaglandin F2alpha
2-AG	glyceryl 2-arachidonate
2,3-dinor-8-iso-PGF1a	2,3-dinor-8-iso-prostaglandin 1alpha
2,3-dinor-8-iso-PGF2a	2,3-dinor-8-iso-prostaglandin F2alpha
4-HDoHE	4-hydroxy-docosahexaenoic acid
5-HEPE	5-hydroxy-eicosapentaenoic acid
5-HETE	5-hydroxy-eicosatetraenoic acid
5-HpEPE	5-hydroxy-eicosatetraenoic acid
5-HpETE	5-hydroperoxy-eicosatetraenoic acid
5-iPF2a-VI	5-iso-prostaglandin F2alpha-VI
5-KETE	5-keto-eicosatetraenoic acid
5,15-DiHEPE	5,15-dihydroxy eicosapentaenoic acid
5,15-DiHETE	5,15-dihydroxy-eicosatetraenoic acid
5,6-DHET	5,6-dihydroxy-eicosatrienoic acid
5,6-DHET-lactone	5,6-dihydroxy-eicosatrienoic acid lactone
5,6-DiHETE	5,6-dihydroxy-eicosatetraenoic acid
5,6-EET	5,6-epoxy-eicosatrienoic acid
5,6-EET-EA	5,6-epoxy-eicosatrienoic acid ethanolamide
5,6-EpETE	5,6-epoxy-eicosatetraenoic acid
5,6-epoxy-18-HEPE	5,6-epoxy-18-hydroxy-eicosapentaenoic acid
5S,14R-LXB4	5,6-epoxy-eicosatetraenoic acid
5S,6R-LXA4	5S,6R-lipoxin A4
5S,6S-LXA4	5S,6S-lipoxin A4
6-keto-PGF1a	6-keto-prostaglandin F1alpha
6-trans-12-epi-LTB4	6-trans-12-epi-leukotriene B4
6-trans-LTB4	6-trans-leukotriene B4
6,15-diketo-13,14-dihydro-PGF1a	6,15-diketo-13,14-dihydro-prostaglandin F1alpha
7-HDoHE	7-hydroxy-docosahexaenoic acid
8-HDoHE	8-hydroxy-docosahexaenoic acid
8-HEPE	8-hydroxy-eicosapentaenoic acid
8-HETE	8-hydroxy-eicosatetraenoic acid
8-iso-13,14-dihydro-15-keto-PGF2a	8-iso-13,14-dihydro-15-keto-prostaglandin F2alpha
8-iso-15-keto-PGF2a	8-iso-15-keto-prostaglandin F2alpha
8-iso-15(R)-PGF2a	8-iso-15(R)-prostaglandin F2alpha
8-iso-PGA1	8-iso-prostaglandin A1
8-iso-PGA2	8-iso-prostaglandin A2
8-iso-PGE1	8-iso-prostaglandin E1
8-iso-PGE2	8-iso-prostaglandin E2
8-iso-PGF1a	8-iso-prostaglandin F1alpha
8-iso-PGF2a	8-iso-prostaglandin F2alpha
8-iso-PGF3a	8-iso-prostaglandin F3alpha
8-iso-PGH2	8-iso-prostaglandin H2
8,12-iso-iPF2a-VI	8,12-iso-isoprostane F2 alpha-VI
8,12-iso-iPF2a-VI-1,5-lactone	8,12-iso-isoprostane F2 alpha-VI-1,5-lactone
8,15-DiHEPE	8,15-dihydroxy eicosapentaenoic acid
8,15-DiHETE	8,15-dihydroxy-eicosatetraenoic acid
8,18-DiHEPE	8,18-dihydroxy-eicosapentaenoic acid
8,9-DHET	8,9-dihydroxy-eicosatrienoic acid
8,9-EET	8,9-epoxy-eicosatrienoic acid
8,9-EET-EA	8,9-epoxy-eicosatrienoic acid ethanolamide
8,9-EpETE	8,9-epoxy-eicosatetraenoic acid
9-HEPE	9-hydroxy-eicosapentaenoic acid
9-HETE	9-hydroxy-eicosatetraenoic acid
9-HODE	9-hydroxy-octadecadienoic acid
9-HOTrE	9-hydroxy-octadecatrienoic acid
9-HpODE	9-hydroperoxy-octadecadienoic acid
9-KODE	9-oxo-octadecadienoic acid
9,10-DiHOME	9,10-dihydroxy-octadecenoic acid
9,10-EpOME	9,10-epoxy-octadecenoic acid
10-HDoHE	10-hydroxy-docosahexaenoic acid
10(S),17(S)-DiHDoHE	10,17-dihydroxy-docosahexaenoic acid
11-beta-13,14-dihydro-15-keto-PGF2a	11-beta-13,14-dihydro-15-keto-prostaglandin F2alpha
11-beta-PGF2a	11-beta-prostaglandin F2alpha
11-dehydro-TXB2	11-dehydro-thromboxane B2
11-HDoHE	11-hydroxy-docosahexaenoic acid
11-HEPE	11-hydroxy-eicosapentaenoic acid
11-HETE	11-hydroxy-eicosatetraenoic acid
11-trans-LTC4	11-trans-leukotriene C4
11-trans-LTE4	11-trans-leukotriene E4
11,12-DHET	11,12-dihydroxy-eicosatrienoic acid
11,12-EET	11,12-epoxy-eicosatrienoic acid
11,12-EET-EA	11,12-epoxy-eicosatrienoic acid ethanolamide
11,12-EpETE	11,12-epoxy-eicosatetraenoic acid
11,18-DiHEPE	11,18-dihydroxy-eicosapentaenoic acid
12-HEPE	12-hydroxy-eicosapentaenoic acid
12-HETE	12-hydroxy-eicosatetraenoic acid
12-HHT	12-hydroxy-heptadecatrienoic acid
12-HpEPE	12-hydroperoxy-eicosapentaenoic acid
12-HpETE	12-hydroperoxy-eicosatetraenoic acid
12-KETE	12-oxo-eicosatetraenoic acid
12-keto-LTB4	12-keto-leukotriene B4
12-OH-17,18-EpETE	12-OH-17,18-epoxyeicosatetraenoic acid
12,13-DiHOME	12,13-dihydroxy-octadecenoic acid
12,13-EpOME	12,13-epoxy-octadecenoic acid
12,18-DiHEPE	12,18-dihydroxy-eicosapentaenoic acid
13-HDoHE	13-hydroxy-docosahexaenoic acid
13-HODE	13-hydroxy-octadecadienoic acid
13-HOTrE	13-hydroxy-octadecatrienoic acid
13-HpODE	13-hydroperoxy-octadecadienoic acid
13-KODE	13-keto-octadecadienoic acid
13,14-dihydro-15-keto-PGD2	13,14-dihydro-15-keto-prostaglandin D2
13,14-dihydro-15-keto-PGE2	13,14-dihydro-15-keto-prostaglandin E2
13,14-dihydro-15-keto-PGF1a	13,14-dihydro-15-keto-prostaglandin F1alpha
13,14-dihydro-15-keto-PGF2a	13,14-dihydro-15-keto-prostaglandin F2alpha
13,14-dihydro-15-keto-PGJ2	13,14-dihydro-15-keto-prostaglandin J2
13,14-dihydro-15-keto-tetranor-PGD2	13,14-dihydro-15-keto-tetranor-prostaglandin D2
13,14-dihydro-15-keto-tetranor-PGE2	13,14-dihydro-15-keto-tetranor-prostaglandin E2
13,14-dihydro-15-keto-tetranor-PGF1a	13,14-dihydro-15-keto-tetranor-prostaglandin F1alpha
13,14-dihydro-15-keto-tetranor-PGF1b	13,14-dihydro-15-keto-tetranor prostaglandin F1beta
13,14-dihydro-15-keto-tetranor-PGF2a	13,14-dihydro-15-keto-tetranor-prostaglandin F2alpha
14-HDoHE	14-hydroxy-docosahexaenoic acid
14,15-DHET	14,15-dihydroxy-eicosatrienoic acid
14,15-DiHETE	14,15-dihydroxy-eicosatetraenoic acid
14,15-EET	14,15-epoxy-eicosatrienoic acid
14,15-EET-EA	14,15-epoxy-eicosatrienoic acid ethanolamide
14,15-EpETE	14,15-epoxy-eicosatetraenoic acid
14,15-LTA4	14,15-leukotriene A4
14,15-LTC4	14,15-leukotriene C4
14,15-LTD4	14,15-leukotriene D4
14,15-LTE4	14,15-leukotriene E4
15-deoxy-delta-12,14-PGJ2	15-deoxy-delta-12,14-prostaglandin J2
15-HEDE	15-hydroxy-eicosadienoic acid
15-HEPE	15-hydroxy-eicosapentaenoic acid
15-HETE	15-hydroxy-eicosapentaenoic acid
15-HETrE	15-hydroxy-eicosatrienoic acid
15-HpEPE	15-hydroperoxy-eicosapentaenoic acid
15-HpETE	15-hydroperoxy-eicosatetraenoic acid
15-KEDE	15-oxo-eicosadienoic acid
15-KETE	15-oxo-eicosatetraenoic acid
15-keto-PGD2	15-keto-prostaglandin D2
15-keto-PGE2	15-keto-prostaglandin E2
15-keto-PGF1a	15-keto-prostaglandin F1alpha
15-keto-PGF2a	15-keto-prostaglandin F2alpha
16-HDoHE	16-hydroxy-docosahexaenoic acid
16-HETE	16-hydroxy-eicosatetraenoic acid
17-HDoHE	17-hydroxy-docosahexaenoic acid
17-HETE	17-hydroxy-eicosatetraenoic acid
17,18-DiHETE	17,18-dihydroxy-eicosatetraenoic acid
17,18-EpETE	17,18-epoxy-eicosatetraenoic acid
18-carboxy-dinor-LTB4	18-carboxy-dinor-leukotriene B4
18-HEPE	18-hydroxy-eicosapentaenoic acid
18-HETE	18-hydroxyicosa-tetraenoic acid
19-HEPE	19-hydroxy-eicosapentaenoic acid
19-HETE	19-hydroxy-eicosatetraenoic acid
19-HETE-EA	19-hydroxyepoxy-eicosatrienoic acid ethanolamide
19,20-DiHDPA	19,20-dihydroxy-docosapentaenoic acid
19,20-EDP	19,20-epoxy-docosapentaenoic acid
20-carboxy-AA	20-carboxy-arachidonic acid
20-carboxy-LTB4	20-carboxy-leukotriene B4
20-HDoHE	20-hydroxy-docosahexaenoic acid
20-HEPE	20-hydroxyeicosa-pentaenoic acid
20-HETE	20-hydroxy-eicosatetraenoic acid
20-HETE-EA	20-hydroxyepoxy-eicosatrienoic acid ethanolamide
20-hydroxy-LTB4	20-hydroxy-leukotriene B4
20-hydroxy-PGE2	20-hydroxy-prostaglandin E2
20-hydroxy-PGF2a	20-hydroxy-prostaglandin F2alpha

Supplementary Table 2

Compoud	Category
AA	AA
iPF2a-IV	AA
LTB4	AA
LTC4	AA
LTD4	AA
LTE4	AA
LTF4	AA
N-acetyl-LTE4	AA
PGA2	AA
PGB2	AA
PGD2	AA
PGE2	AA
PGF2a	AA
PGJ2	AA
PGK2	AA
tetranor-PGDM	AA
tetranor-PGEM	AA
tetranor-PGFM	AA
TXB2	AA
2,3-dinor-8-iso-PGF2a	AA
5-HETE	AA
5-HpETE	AA
5-iPF2a-VI	AA
5-KETE	AA
5,6-DHET	AA
5,6-DHET-lactone	AA
5,6-EET	AA
5S,6R-LXA4	AA
5S,6S-LXA4	AA
5S,14R-LXB4	AA
5,15-DiHETE	AA
6-keto-PGF1a	AA
6-trans-LTB4	AA
6,15-diketo-13,14-dihydro-PGF1a	AA
8-HETE	AA
8-iso-13,14-dihydro-15-keto-PGF2a	AA
8-iso-15-keto-PGF2a	AA
8-iso-15(R)-PGF2a	AA
8-iso-PGA2	AA
8-iso-PGE2	AA
8-iso-PGF2a	AA
8,9-DHET	AA
8,9-EET	AA
8,12-iso-iPF2a-VI-1,5-lactone	AA
8,15-DiHETE	AA
9-HETE	AA
11-beta-13,14-dihydro-15-keto-PGF2a	AA
11-beta-PGF2a	AA
11-dehydro-TXB2	AA
11-HETE	AA
11-trans-LTC4	AA
11-trans-LTE4	AA
11,12-DHET	AA
11,12-EET	AA
12-HETE	AA
12-HHT	AA
12-HpETE	AA
12-KETE	AA
12-keto-LTB4	AA
13,14-dihydro-15-keto PGJ2	AA
13,14-dihydro-15-keto-PGD2	AA
13,14-dihydro-15-keto-PGE2	AA
13,14-dihydro-15-keto-PGF2a	AA
13,14-dihydro-15-keto-tetranor-PGD2	AA
13,14-dihydro-15-keto-tetranor-PGE2	AA
13,14-dihydro-15-keto-tetranor-PGF1b	AA
14,15-DHET	AA
14,15-EET	AA
14,15-LTC4	AA
14,15-LTE4	AA
15-deoxy-delta-12,14-PGJ2	AA
15-HETE	AA
15-HpETE	AA
15-keto-PGE2	AA
15-keto-PGF2a	AA
16-HETE	AA
17-HETE	AA
18-carboxy-dinor-LTB4	AA
18-HETE	AA
19-HETE	AA
20-carboxy-AA	AA
20-carboxy-LTB4	AA
20-HETE	AA
20-hydroxy-LTB4	AA
20-hydroxy-PGE2	AA
20-hydroxy-PGF2a	AA

Compoud	Category
1a1b-dihomo-PGF2a	ADA
9-HOTrE	ALA
13-HOTrE	ALA
PGA1	DGLA
PGD1	DGLA
PGE1	DGLA
TXB1	DGLA
8-iso-PGA1	DGLA
8-iso-PGE1	DGLA
8-iso-PGF1a	DGLA
13,14-dihydro-15-keto-tetranor-PGF1a	DGLA
15-HETrE	DGLA
DHA	DHA
Maresin1	DHA
Resolvin D1	DHA
Resolvin D2	DHA
4-HDoHE	DHA
7-HDoHE	DHA
7,17-hydroxy-DPA	DHA
8-HDoHE	DHA
10-HDoHE	DHA
10,17-DiHDoHE	DHA
11-HDoHE	DHA
13-HDoHE	DHA
14-HDoHE	DHA
16-HDoHE	DHA
17-HDoHE	DHA
20-HDoHE	DHA
AEA	EA
LTB4-EA	EA
OEA	EA
PGD2-EA	EA
PGE1-EA	EA
PGE2-EA	EA
PGF2a-EA	EA
11,12-EET-EA	EA
14,15-EET-EA	EA
5,6-EET-EA	EA
8,9-EET-EA	EA
15-HEDE	EDA
15-KEDE	EDA
EPA	EPA
LXA5	EPA
PGD3	EPA
PGE3	EPA
PGF3a	EPA
TXB3	EPA
5-HEPE	EPA
5-HpEPE	EPA
5,6-DiHETE	EPA
8-iso-PGF3a	EPA
12-HEPE	EPA
12-HpEPE	EPA
14,15-DiHETE	EPA
15-HEPE	EPA
15-HpEPE	EPA
17,18-DiHETE	EPA
17,18-EpETE	EPA
18-HEPE	EPA
9-HODE	LA
9-HpODE	LA
9-KODE	LA
9,10-DiHOME	LA
9,10-EpOME	LA
12,13-DiHOME	LA
12,13-EpOME	LA
13-HODE	LA
13-HpODE	LA
13-KODE	LA
Azelaoyl-PAF	PAF
Lyso-PAF	PAF
PAF	PAF

Supplementary Table 3

Compoud	category	Saline peak intensity	polyI:C peak intensity	polyI:C/Saline log2FC	polyI:C/Saline Pvalue
AA	AA	2.4583 ± 0.0942	2.2130 ± 0.05940	***-0.152***	***0.043775***
LTB4	AA	0.0032 ± 0.0009	0.0097 ± 0.00315	1.628	0.074824
LTC4	AA	0.0009 ± 0.0002	0.0064 ± 0.00209	***2.817***	***0.029661***
LTE4	AA	0.0293 ± 0.0033	0.0439 ± 0.00938	0.586	0.171252
PGA2	AA	0.0218 ± 0.0039	0.0457 ± 0.01980	1.069	0.267267
PGD2	AA	0.0088 ± 0.0017	0.0314 ± 0.00630	***1.841***	***0.006876***
PGE2	AA	0.0134 ± 0.0039	0.0896 ± 0.06715	2.740	0.289681
PGF2a	AA	0.0161 ± 0.0038	0.0271 ± 0.00674	0.750	0.179840
PGJ2	AA	0.0146 ± 0.0033	0.0245 ± 0.00819	0.747	0.287320
tetranor-PGDM	AA	0.0298 ± 0.0050	0.2884 ± 0.05008	***3.273***	***0.000834***
tetranor-PGEM	AA	0.0190 ± 0.0044	0.1589 ± 0.01263	***3.067***	***0.000001***
TXB2	AA	0.0212 ± 0.0077	0.0876 ± 0.05302	2.044	0.249257
5-HETE	AA	0.2896 ± 0.0218	0.2951 ± 0.02403	0.027	0.868848
5-KETE	AA	0.0007 ± 0.0001	0.0006 ± 0.00010	-0.230	0.480527
5,6-DHET	AA	0.0120 ± 0.0018	0.0117 ± 0.00201	-0.038	0.907611
6-keto-PGF1a	AA	0.1331 ± 0.0306	0.2831 ± 0.07616	1.089	0.096030
6,15-diketo-13,14-dihydro-PGF1a	AA	0.0551 ± 0.0159	0.1074 ± 0.02989	0.964	0.147416
8-HETE	AA	0.0419 ± 0.0048	0.0385 ± 0.00993	-0.123	0.762805
8-iso-PGE2	AA	0.0032 ± 0.0004	0.0043 ± 0.00078	0.405	0.254525
8,9-DHET	AA	0.0281 ± 0.0019	0.0325 ± 0.00723	0.210	0.570419
8,12-iso-iPF2a-VI-1,5-lactone	AA	0.0217 ± 0.0041	0.0284 ± 0.00475	0.391	0.299827
9-HETE	AA	0.0332 ± 0.0066	0.0277 ± 0.00558	-0.262	0.532423
11-HETE	AA	0.0540 ± 0.0039	0.1343 ± 0.05783	1.315	0.202916
11,12-DHET	AA	0.0443 ± 0.0026	0.0492 ± 0.01094	0.152	0.672049
12-HETE	AA	0.2600 ± 0.0566	0.6349 ± 0.48095	1.288	0.460525
13,14-dihydro-15-keto-PGD2	AA	0.0030 ± 0.0004	0.0068 ± 0.00275	1.200	0.200455
13,14-dihydro-15-keto-PGE2	AA	0.0036 ± 0.0007	0.0648 ± 0.04334	4.167	0.195499
14,15-DHET	AA	0.1334 ± 0.0069	0.1180 ± 0.00860	-0.177	0.180648
15-HETE	AA	0.0434 ± 0.0040	0.0549 ± 0.01290	0.340	0.414274
17-HETE	AA	0.0112 ± 0.0014	0.0106 ± 0.00216	-0.074	0.831703
18-HETE	AA	0.0457 ± 0.0037	0.0399 ± 0.00592	-0.196	0.419146
19-HETE	AA	0.0521 ± 0.0052	0.0363 ± 0.00478	***-0.519***	***0.039603***
9-HOTrE	ALA	0.0177 ± 0.0014	0.0127 ± 0.00212	-0.486	0.066292
13-HOTrE	ALA	0.0275 ± 0.0017	0.0246 ± 0.00378	-0.162	0.497282
PGE1	DGLA	0.0010 ± 0.0000	0.0012 ± 0.00012	0.153	0.346594
15-HETrE	DGLA	0.0742 ± 0.0093	0.0563 ± 0.01315	-0.400	0.281867
DHA	DHA	49.4411 ± 2.0103	39.5040 ± 2.36811	***-0.324***	***0.005518***
4-HDoHE	DHA	0.4422 ± 0.0339	0.2958 ± 0.02942	***-0.580***	***0.004637***
7-HDoHE	DHA	0.1424 ± 0.0135	0.1453 ± 0.02791	0.028	0.928751
8-HDoHE	DHA	0.2885 ± 0.0227	0.3118 ± 0.07608	0.112	0.775794
10-HDoHE	DHA	0.1086 ± 0.0087	0.0985 ± 0.01169	-0.142	0.495708
11-HDoHE	DHA	0.1688 ± 0.0097	0.1319 ± 0.01594	-0.356	0.068615
13-HDoHE	DHA	0.0482 ± 0.0054	0.0399 ± 0.00455	-0.270	0.258550
14-HDoHE	DHA	0.1777 ± 0.0306	0.1297 ± 0.02690	-0.454	0.255574
16-HDoHE	DHA	0.1111 ± 0.0094	0.1014 ± 0.00754	-0.131	0.433638
17-HDoHE	DHA	0.0314 ± 0.0033	0.0238 ± 0.00419	-0.400	0.171820
20-HDoHE	DHA	0.0943 ± 0.0051	0.0683 ± 0.00516	***-0.466***	***0.002236***
AEA	EA	0.0296 ± 0.0010	0.0289 ± 0.00216	-0.033	0.781035
OEA	EA	0.8030 ± 0.0245	0.7013 ± 0.02687	***-0.195***	***0.012635***
15-KEDE	EDA	0.0038 ± 0.0003	0.0033 ± 0.00027	-0.189	0.283460
EPA	EPA	2.4065 ± 0.1255	1.8300 ± 0.11654	***-0.395***	***0.003674***
5-HEPE	EPA	0.1321 ± 0.0101	0.0964 ± 0.00940	***-0.455***	***0.018967***
5,6-DiHETE	EPA	0.0216 ± 0.0016	0.0180 ± 0.00272	-0.267	0.268515
12-HEPE	EPA	0.0955 ± 0.0205	0.0835 ± 0.03629	-0.193	0.779326
14,15-DiHETE	EPA	0.0971 ± 0.0085	0.1119 ± 0.02864	0.204	0.632364
15-HEPE	EPA	0.1214 ± 0.0110	0.0869 ± 0.00953	***-0.483***	***0.030168***
17,18-DiHETE	EPA	0.2843 ± 0.0197	0.3523 ± 0.10637	0.309	0.545891
18-HEPE	EPA	0.0814 ± 0.0069	0.0623 ± 0.00887	-0.386	0.108635
9-HODE	LA	0.4210 ± 0.0266	0.3023 ± 0.03110	***-0.478***	***0.010271***
9-KODE	LA	0.0505 ± 0.0071	0.0406 ± 0.00744	-0.314	0.351568
9,10-DiHOME	LA	7.0738 ± 0.4437	5.4276 ± 0.75189	-0.382	0.081670
12,13-DiHOME	LA	7.6443 ± 0.5574	5.8861 ± 0.90402	-0.377	0.120877
13-HODE	LA	1.8456 ± 0.1328	1.2792 ± 0.12717	***-0.529***	***0.006785***
13-KODE	LA	0.1596 ± 0.0169	0.1117 ± 0.01761	-0.514	0.067007
Azelaoyl-PAF	PAF	0.1009 ± 0.0138	0.0968 ± 0.00924	-0.060	0.806307
Lyso-PAF	PAF	818.4672 ± 18.0827	793.2767 ± 30.34829	-0.045	0.488213
